# Selecting patient-reported outcome measures for a patient-facing technology

**DOI:** 10.1093/jamiaopen/ooad104

**Published:** 2023-12-13

**Authors:** Priyank Raj, Youmin Cho, Yun Jiang, Yang Gong

**Affiliations:** D. Bradley McWilliams School of Biomedical Informatics, The University of Texas Health Science Center at Houston, Houston, TX 77030, United States; D. Bradley McWilliams School of Biomedical Informatics, The University of Texas Health Science Center at Houston, Houston, TX 77030, United States; School of Nursing, University of Michigan, Ann Arbor, MI 48109, United States; D. Bradley McWilliams School of Biomedical Informatics, The University of Texas Health Science Center at Houston, Houston, TX 77030, United States

**Keywords:** patient-reported outcome measure, breast cancer, patient-facing technology, symptoms, quality of life

## Abstract

**Objective:**

This article provides insight into our process and considerations for selecting patient-reported outcome measures (PROMs) designed for self-reporting symptoms and quality-of-life among breast cancer (BCA) patients undergoing oral anticancer agent treatment via a patient-facing technology (PFT) platform.

**Methods:**

Following established guidelines, we conducted a thorough assessment of a specific set of PROMs, comparing their content to identify the most suitable options for studying BCA patients.

**Results:**

We recommend utilizing the combination of EORTC QLQ-C30 + EORTC QLQ-BR45 as the preferred instrument, especially when developing a dedicated “breast cancer-only” application.

**Discussion:**

When developing and maintaining a dashboard for a PFT platform that includes multiple cancer types, it is important to consider the feasibility of interface design and workload. To achieve this, we recommend using PRO-CTCAE+PROMIS 10 GH for the PFT. Moreover, it is important to consider adding ad hoc items to complement the chosen PROM(s).

**Conclusion:**

This article describes our efforts to identify PROMs for self-reported data while considering patient and developer burdens, providing guidance to PFT developers facing similar challenges in PROM selection.

## Background and significance

Patient-reported outcome measures (PROMs) are standardized questionnaires that patients use to report their health status, particularly relevant for cancer patients undergoing treatment to document cancer-related symptoms and treatment side effects. Patient-facing technologies (PFTs), frequently integrating electronic patient-reported outcomes (ePRO), aim to improve patient engagement, enhance patient experience, facilitate self-care, and ultimately contribute to better health outcomes.^1^ These technologies have gained growing importance in healthcare, with the emergence of desktop, mobile, and web applications demonstrating notable benefits. During our efforts to develop a PFT to assist patients with breast, lung, prostate, and colorectal cancer, we faced the challenge of choosing the most appropriate PROM that could efficiently assess patients’ health status while minimizing the reporting burden. Additionally, creating a dashboard to display PROM data and depict the evolving trends in PROs over time^2^ proved to be a complex task, intended to enhance patient self-assessment and communication with healthcare providers.^3^ Furthermore, this PFT is designed to adapt and generate personalized, magazine-style patient education and self-management suggestions based on PROs.

Existing literature on integrating PROMs into routine cancer care revealed a lack of clear guidelines or standards for PROM selection.^4–6^ This article adopts a systematic approach to compare prominent PROMs designed for breast cancer (BCA). Additionally, we delve into the complexities of PROM selection, discuss the strengths and weaknesses of the PROMs we have chosen, and explore potential avenues for future research on PFTs that support multiple cancer types. Using BCA PROMs as a case study, this article aims to provide insights for fellow researchers and PFT developers encountering comparable difficulties in PROM selection.

## Objectives

The primary goals of this study were to determine the most appropriate PROMs for self-reported symptom assessment and quality of life evaluation among BCA patients receiving home-based care through a PFT. Additionally, we explored the reporting burden and the feasibility of design considerations for a broader range of cancer types.

## Materials and methods

### Patient population group

Our focus is on adult BCA patients receiving home-based care, especially those undergoing treatments such as oral anticancer agents (OAAs). These patients exhibit an adequate performance status that allows them to actively engage in self-reporting. Furthermore, they have experienced a shift in their care from medical facilities to home-based settings within the past year. The selected PROMs/items must proficiently track and evaluate the symptoms associated with their particular treatment(s).

### Principles for PROM selection

To systematically select the most appropriate PROMs for our application, we adopted the 6 principles proposed by Luckett et al.^7^ The first principle states that the population characteristics should be carefully considered in PROM selection.^7^ The second is to use proximal PROs (such as symptoms) as the primary outcome and distal PROs (such as QoL) as the secondary outcome.^7^ The third and fourth principles state that PROMs should be identified primarily based on the content and structure of subscales within the PROM, and they should be supported by the adequacy of evidence for psychometric properties (such as reliability and validity) and track records of use in similar populations. It should be noted that psychometric evidence must be documented for the ePRO mode of administration.^8^ The fifth principle discusses feasibility regarding respondent burden, cost, availability of translations, etc., while the final one states that *ad hoc* or supplementary items must be used minimally and can be appended at the end of the main PROMs and should be scored separately.^7^

### Major PROMs for consideration

Many published questionnaires are available for measuring symptoms and quality of life in cancer patients, including PROMs specific to BCA.^4^ A comprehensive list can be found on the PROQOLID (Patient-Reported Outcome and Quality of Life Instruments Database) database (accessed July 21, 2023, https://www.qolid.org/). Further, several review articles have compared the PROMs most widely used in BCA.^4–6^ A comprehensive list of PROMs used in BCA studies has been listed in Appendix SA. Amongst the select PROMs, the QLQ-BR23/BR45 and FACT-B are the only 2 quality-of-life instruments explicitly developed for BCA patients “facing different disease stages and treatments”.^4^ Both are widely used in multiple BCA treatments and act as supplements to their general cancer PROMs, namely the EORTC QLQ-C30 and FACT-G. EORTC QLQ-C30 is a 30-item cancer-specific QoL instrument with items on function, global QoL, and symptoms.^6^ The BR23/BR45 addresses several concerns specific to BCA.^9^ FACT-G is also a cancer-specific QoL instrument with 4 well-being subscales and includes one item on global QoL. Its BCA supplement FACT-B (which, unlike BR23, subsumes the FACT-G) has an additional scale covering items of concern specific to BCA.^10^ Another highlighted instrument is the Patient-Reported Outcome—Common Terminology Criteria for Adverse Events (PRO-CTCAE). PRO-CTCAE has been the gold standard instrument for reporting symptomatic adverse events in many cancer types for more than 30 years and provides a comprehensive assessment of cancer-related symptoms through a library containing 124 items representing 78 adverse events, divided into 14 categories by organ system.^11^

Other prominent PROMS include BREAST-Q and PROMIS, which we have reviewed but excluded for further consideration. BREAST-Q is focused on breast surgery patients instead of the BCA population. The PROMIS suite lacks a BCA-specific module. Nonetheless, PROMIS 10 SF could be used as a QoL PROM. We also reviewed the BCA PROM recommendations made by 2 consortia, namely International Consortium for Health Outcome Measures (ICHOM),^12^ and Translational Breast Cancer Research Consortium (TBCRC).^13^ We have verified that our PROMs cover the core outcome set described in the recommendations.

In summary, we examined the 3 PROMs mentioned above (EORTC QLQ-C30+BR45, FACT-B, and PRO-CTCAE) and conducted a comparative analysis to identify the most suitable option(s) for our application, complemented by *ad hoc* items.

## Results

### Item-wise comparison of selected PROMs

Table 1 provides a detailed comparison of the selected instruments (namely, the PRO-CTCAE, C30+BR45, and FACT-B) by items covered, and the interpretation is discussed in the next section. The latter 2 instruments also measure function/ well-being, and QoL. EORTC-CIPN20 and FACT-ES have also been listed to support the discussion on *ad hoc* items later.

**Table 1.** Selected PROMs and their outcome coverage.

	1	2		3	*Ad hoc*	
Outcomes vs PROMs	PRO-CTCAE	C30	BR45	FACT-B	CIPN20	FACT-ES
Oral symptoms	✓	X	✓	X	X	X
Gastrointestinal symptoms	✓	✓	✓	✓	X	✓
Respiratory symptoms	✓	✓	X	✓	X	X
Cardio/circulatory symptoms	✓	X	X	X	X	X
Cutaneous symptoms	✓	X	✓	✓	X	X
Neurological symptoms	✓	X	✓	X	✓	X
Visual/perceptual	✓	X	✓	X	✓	X
Attention/memory	✓	✓	X	X	X	X
Pain	✓	✓	✓	✓	X	✓
Sleep/wake	✓	✓	X	✓	X	✓
Mood	✓	✓	✓	✓	X	✓
Genito-urinary symptoms	✓	X	X	X	X	X
Sexual	✓	X	✓	✓	✓	✓
Vaginal symptoms	✓	X	✓	X	X	✓
Vasomotor symptoms (hot flashes and sweating)	✓	X	✓	X	X	✓
Arm symptoms	X	X	✓	✓	X	X
Breast symptoms	✓	X	✓	X	X	✓
Weight gain	X	X	✓	✓	X	✓
Body image	X	X	✓	✓	X	X
Financial toxicity	X	✓	X	X	X	X
Function	X	✓	X	✓	✓	✓
Global QoL	X	✓	X	✓	X	✓

Note: Highlighted portions indicate the items we would include in a BCA application battery.

### Instrument of choice

The PRO-CTCAE provides a comprehensive library of cancer-specific symptomatic adverse events; however, in its entirety, the PRO-CTCAE raises feasibility issues when considering respondent burden due to its large size. Not all items are relevant to every cancer type, and a customized selection of items is recommended by both PRO-CTCAE developers^11^ and researchers.^14^ Furthermore, several concerns pertinent to BCA, such as arm and breast symptoms, weight gain, and body image, are not included in the PRO-CTCAE. On the other hand, the BR45 and FACT-B are shorter instruments that were developed keeping BCA disease and treatment in mind and thus provide a tailored collection of BCA-relevant symptoms, including items not covered by the PRO-CTCAE.

Thus, we recommend the use of EORTC QLQ-C30 + EORTC QLQ-BR45 as the primary instrument for symptom and quality-of-life reporting for a PFT specific for BCA, preferred over FACT-B due to its more inclusive content. *Ad hoc* items may be derived from the PRO-CTCAE, EORTC QLQ-CIPN20, FACT-ES, and patient free-text entry, based on provider/researcher suggestions, as discussed below.

When expanding the PFT design beyond BCA, we chose to integrate PRO-CTCAE into our PFT, guided by the fifth principle of feasibility regarding respondent burden, cost, etc. The logic behind this choice stems from our application’s user base, which encompasses patients with 4 distinct cancer types. Employing separate instruments for each cancer type would impose a substantial workload on developers tasked with designing customized dashboards. To simplify and streamline this process, we opted for the straightforward symptom severity items derived from PRO-CTCAE, which can be universally applied across all cancer types.

In our research, we discovered that the most suitable instrument might vary depending on the cancer type. For instance, BR45 proves effective for BCA while FACT-P works well for advanced prostate cancer. Implementing specific instruments for each cancer type would entail additional efforts for developers in designing tailored dashboards. Since PRO-CTCAE focuses solely on symptom assessment and does not measure QoL, we included PROMIS 10 Short Form in our battery of measures. This addition includes 2 items pertaining to global QoL to ensure a comprehensive evaluation of patients’ well-being.

### Principles applied for the instrument selection

Both the PRO-CTCAE and the EORTC instruments follow the first 4 principles in that they are relevant to the patient population group and measure symptoms as primary outcome. The third principle is also satisfied in the EORTC and PRO-CTCAE batteries, in that symptoms are measured followed by QoL. Regarding the fourth principle, evidence for psychometric properties (reliability, validity, and responsiveness) supporting all the instruments exist moderately to extensively for paper versions but is more limited for the ePRO version.^8^^,^^15–20^ Using the C30+BR45 in its entirety rather than selecting items prevents possible jeopardization of the construct validity of established scales of the instrument, honoring the third and fourth principles.

Patient burden is a crucial factor, as extensive questionnaires often lead to missing values due to nonresponses. It is generally recommended that a survey should require no more than 10-20 min^11^ for patients to complete, equating to approximately 100 items or less. The C30+BR45 survey (*N* = 75) falls within this limit. A similar outcome can be achieved with PRO-CTCAE+PROMIS 10 SF (*N* = 134) if we utilize a subset of PRO-CTCAE, which we intend to do in our study. All the instruments listed in Table 1 have free permission for use, except the chemotherapy-induced peripheral neuropathy (CIPN)-20.

### *Ad hoc* items

Assuming QLQ-C30 + BR23/45 as the primary instrument, there are multiple sources of *ad hoc* items, which can be added/deleted from the main battery throughout the period of patient enrollment. First, we suggest adding the 2 cardio/circulatory items of the PRO-CTCAE to track heart failure. It is now recognized that there is an increased risk of heart failure in BCA patients due to the effects of treatment and the overlap of risk factors between the 2 diseases.^21^ Other PRO-CTCAE symptom items not extensively covered by C30/BR45 (such as cutaneous and genitourinary items) may be offered as a checkbox-style library for selection by the patients during each survey, and the items selected by individual patients can be added to the personalized batteries in subsequent surveys. Secondly, we recommend employing the EORTC QLQ-CIPN20^22^ as a tool to assess sensory, autonomic, and motor peripheral neuropathy in patients undergoing treatment with specific chemotherapeutic agents, notably taxanes, which are recognized for their potential to induce peripheral neuropathy.

Peripheral neuropathy is a common toxicity observed in long-term BCA survivors treated with taxane-based chemotherapy.^23^ It is worth mentioning that the PRO-CTCAE includes just 2 items related to sensory neuropathy. Similarly, patients treated with oral endocrine therapy may present unique vaginal symptoms such as vaginal discomfort and pain or dryness vagina during sexual activity not tracked by the BR45. For such symptoms, items from the FACT-Endocrine Symptoms (ES) may be used as *ad hoc* items.^13^ Similar suggestions may be made for mental health and a more granular assessment of pain and other symptom clusters. Last but not least, patients should have the option to make free-text entries to report their concerns through the PFT, and such reports can be used to invent more *ad hoc* items for uncommon symptoms in subsequent surveys in an individualized manner. It is important to acknowledge that *ad hoc* items not only add to the developer burden but also raise psychometric concerns. Therefore, we suggest exercising caution and using them sparingly.

## Discussion

The article summarizes a systematic approach to PROM selection for a PFT and recommends a precise battery of instruments for use with BCA patients. Literature on PROMs used for routine cancer care in PFTs often does not describe their criteria for PROM selection or how the selected PROMs/items meet the needs of a heterogeneous patient population. In this article, we have delineated our PROM selection process and how *ad hoc* items can support reporting tasks by a heterogeneous population, that is, patients under chemotherapeutic/hormonal/targeted OAA therapy. Our battery includes the core outcome sets listed in the consortia recommendations. Further research is needed to document ePRO psychometric evidence for the above instruments. Time points of assessment, scoring, and thresholds for clinical alert generation need further discussion.

## Conclusion

We applied the 6 principles to justify our choice of PROMs for BCA patients in a PFT. Combining PROMs such as EORTC QLQ-C30, BR45, and *ad hoc* items can potentially yield comprehensive PRO data encompassing symptoms and QoL from BCA patients. However, opting for PRO-CTCAE in our PFT, which serves 4 different cancer types, proves to be more practical and feasible. The simplicity of symptom-severity items facilitates visualization, tracking, and trend analysis without requiring extensive technical complexities in dashboard design. Additionally, feasibility considerations related to dashboard design can be proposed as an additional subprinciple within the fifth Luckett principle that developers should consider when selecting a PROM. Further research is necessary to explore symptoms associated with OAAs during care transitions. Moreover, there is a need to identify PROMs that can efficiently capture self-reported data while considering the burdens on both patients and developers.

## Supplementary Material

ooad104_Supplementary_Data

## Data Availability

We hereby declare that neither any original data was generated nor was any third-party data analyzed during the course of this study.

## References

[1] Ahern DK , Woods SS, Lightowler MC, Finley SW, Houston TK. Promise of and potential for patient-facing technologies to enable meaningful use. Am J Prev Med. 2011;40(5):S162-S172. 21521591 10.1016/j.amepre.2011.01.005

[2] Singhal U , Skolarus TA, Gore JL, et al Implementation of patient-reported outcome measures into health care for men with localized prostate cancer. Nat Rev Urol. 2022;19(5):263-279. 35260844 10.1038/s41585-022-00575-4

[3] Mooney K , Whisenant MS, Beck SL. Symptom care at home: a comprehensive and pragmatic PRO system approach to improve cancer symptom care. Med Care. 2019;57(Suppl 1):S66-S72. 30531525 10.1097/MLR.0000000000001037 PMC8087999

[4] Salas M , Mordin M, Castro C, Islam Z, Tu N, Hackshaw MD. Health-related quality of life in women with breast cancer: a review of measures. BMC Cancer. 2022;22(1):66. 35033009 10.1186/s12885-021-09157-w PMC8760726

[5] Turner-Bowker DM , Hao Y, Foley C, et al The use of patient-reported outcomes in advanced breast cancer clinical trials: a review of the published literature. Curr Med Res Opin. 2016;32(10):1709-1717. 27331272 10.1080/03007995.2016.1205005

[6] Cardoso F , Cella D, Velikova G, et al Quality-of-life methodology in hormone receptor-positive advanced breast cancer: Current tools and perspectives for the future. *Cancer Treat Rev*. 2022;102:102321. 10.1016/j.ctrv.2021.102321 34852292

[7] Luckett T , King MT. Choosing patient-reported outcome measures for cancer clinical research—practical principles and an algorithm to assist non-specialist researchers. Eur J Cancer. 2010;46(18):3149-3157. 20869232 10.1016/j.ejca.2010.08.002

[8] Pérez-Alfonso KE , Sánchez-Martínez V. Electronic patient-reported outcome measures evaluating cancer symptoms: a systematic review. Semin Oncol Nurs. 2021;37(2):151145. 33773879 10.1016/j.soncn.2021.151145

[9] Bjelic-Radisic V , Cardoso F, Cameron D, et al An international update of the EORTC questionnaire for assessing quality of life in breast cancer patients: EORTC QLQ-BR45. Ann Oncol off J Eur Soc Med Oncol. 2020;31(2):283-288. 10.1016/j.annonc.2019.10.027 31959345

[10] Nguyen J , Popovic M, Chow E, et al EORTC QLQ-BR23 and FACT-B for the assessment of quality of life in patients with breast cancer: a literature review. J Comp Eff Res. 2015;4(2):157-166. 25825844 10.2217/cer.14.76

[11] Basch E , Reeve BB, Mitchell SA, et al Development of the national cancer institute’s patient-reported outcomes version of the common terminology criteria for adverse events (PRO-CTCAE). J Natl Cancer Inst. 2014;106(9):dju244. 25265940 10.1093/jnci/dju244 PMC4200059

[12] Ong WL , Schouwenburg MG, van Bommel ACM, et al A standard set of value-based patient-centered outcomes for breast cancer: the international consortium for health outcomes measurement (ICHOM) initiative. JAMA Oncol. 2017;3(5):677-685. 28033439 10.1001/jamaoncol.2016.4851

[13] Bowen DJ , Shinn EH, Gregrowski S, et al Patient-reported outcomes in the translational breast cancer research consortium. Cancer. 2020;126(5):922-930. 31743427 10.1002/cncr.32615 PMC7063688

[14] Bouazza YB , Chiairi I, El Kharbouchi O, et al Patient-reported outcome measures (proms) in the management of lung cancer: A systematic review. *Lung Cancer*. 2017;113:140-151. 10.1016/j.lungcan.2017.09.011 29110842

[15] Bennett AV , Dueck AC, Mitchell SA, et al; National Cancer Institute PRO-CTCAE Study Group. Mode equivalence and acceptability of tablet computer-, interactive voice response system-, and paper-based administration of the U.S. National Cancer Institute’s patient-reported outcomes version of the common terminology criteria for adverse events (PRO-CTCAE). Health Qual Life Outcomes. 2016;14(1):24. 26892667 10.1186/s12955-016-0426-6 PMC4759776

[16] Wallwiener M , Matthies L, Simoes E, et al Reliability of an e-PRO tool of EORTC QLQ-C30 for measurement of health-related quality of life in patients with breast cancer: prospective randomized trial. J Med Internet Res. 2017;19(9):e322. 28912116 10.2196/jmir.8210 PMC5620457

[17] Velikova G , Wright EP, Smith AB, et al Automated collection of quality-of-life data: a comparison of paper and computer touch-screen questionnaires. J Clin Oncol. 1999;17(3):998-1007. 10071295 10.1200/JCO.1999.17.3.998

[18] Gundy CM , Aaronson NK. Effects of mode of administration (MOA) on the measurement properties of the EORTC QLQ-C30: a randomized study. Health Qual Life Outcomes. 2010;8(1):35. 20353582 10.1186/1477-7525-8-35 PMC2855522

[19] Matthies LM , Taran FA, Keilmann L, et al An electronic patient-reported outcome tool for the FACT-B (Functional Assessment of Cancer Therapy-Breast) questionnaire for measuring the health-related quality of life in patients with breast cancer: reliability study. J Med Internet Res. 2019;21(1):e10004. 30668517 10.2196/10004 PMC6362389

[20] Knoerl R , Gray E, Stricker C, et al Electronic versus paper-pencil methods for assessing chemotherapy-induced peripheral neuropathy. Support Care Cancer. 2017;25(11):3437-3446. 28577231 10.1007/s00520-017-3764-y

[21] Gulati M , Mulvagh SL. The connection between the breast and heart in a woman: breast cancer and cardiovascular disease. Clin Cardiol. 2018;41(2):253-257. 29446841 10.1002/clc.22886 PMC6489752

[22] Cho Y , Ruddy KJ, Smith EM. Evaluation of chemotherapy-induced peripheral neuropathy. In: Lustberg M, Loprinzi C, eds. Diagnosis, Management and Emerging Strategies for Chemotherapy-Induced Neuropathy. A MASCC Book. Cham: Springer; 2021:53-93.

[23] Bao T , Basal C, Seluzicki C, Li SQ, Seidman AD, Mao JJ. Long-term chemotherapy-induced peripheral neuropathy among breast cancer survivors: prevalence, risk factors, and fall risk. Breast Cancer Res Treat. 2016;159(2):327-333. 27510185 10.1007/s10549-016-3939-0 PMC5509538

