# Supplementary material for: Selecting patient-reported outcome measures for a patient-facing technology
Source: JAMIA Open. 2023 Dec 13;6(4):ooad104. doi: 10.1093/jamiaopen/ooad104 (PMC10719077; doi:10.1093/jamiaopen/ooad104)
Supplement: ooad104_Supplementary_Data [file ooad104_supplementary_data.docx]

**MULTITUDE OF PROMs AVAILABLE**

Specific to breast cancer (BCA): BCQ, FACT-B, IBCSG-QLC, QL, SHE, EORTC QLQ-C30 + EORTC QLQ-BR23/45, EQ-5D, BPI-SF, FBSI, NFBSI-16, YW-BCI36, BCSS, QuEST-Br, QLICP-BR, INA-BCHRQoL

Pan-cancer: PRO-CTCAE

**INSTRUMENT OF CHOICE FOR A BCA-ONLY APPLICATION**

- EORTC QLQ-C30 + EORTC QLQ-BR23/45

**REASONS FOR EXCLUDING ALL PROMS SPECIFIC TO BCA EXCEPT THE EORTC AND FACT PROMS:**

The QLQ-BR23/BR45 and FACT-B are the only two quality-of-life instruments explicitly developed for BCA patients “facing different disease stages and treatments” and are amongst the most widely used PROMs in multiple breast cancer treatments.

**3 PROMs SELECTED FOR A FINAL COMPARISON**

- PRO-CTCAE
- EORTC QLQ-C30 + EORTC QLQ-BR23/45
- FACT-B

**INSTRUMENT OF CHOICE FOR A PAN-CANCER APPLICATION**

- PRO-CTCAE + PROMIS 10 GH

**Selection flowchart of Patient Reported Outcome Measures (PROMs) for use in a patient facing technology**
